# Transcriptomic analysis-driven identification and transcriptional characterization of *Komagataella phaffii* promoters from highly transcribed endogenous genes across diverse culture conditions

**DOI:** 10.7717/peerj.21479

**Published:** 2026-07-21

**Authors:** Karla B. Fernández-Cano, José M. Viader-Salvadó, Martha Guerrero-Olazarán

**Affiliations:** Universidad Autónoma de Nuevo León, UANL, Facultad de Ciencias Biológicas, Instituto de Biotecnología, San Nicolás de los Garza, Nuevo León, Mexico

**Keywords:** Methanol-free promoters, *Pichia pastoris*, RNA-seq, RT-qPCR, Transcriptomic analysis, Strong promoter candidates

## Abstract

The yeast *Komagataella phaffii* (formerly *Pichia pastoris*) is a robust host for recombinant protein production, capable of growing at high cell densities, performing post-translational modifications, and efficiently secreting heterologous proteins. However, gene expression still relies mainly on the methanol-inducible *AOX1* promoter (P_AOX1_) and the constitutive *GAPDH* promoter (P_GAP_), highlighting the need for alternative promoters with distinct regulatory properties. This study aimed to identify and characterize promoters from the most highly transcribed endogenous genes across diverse culture conditions as strong promoter candidates to expand the genetic toolbox for this yeast. Seven promoters were identified through RNA-seq analysis from genes showing transcript levels comparable to or higher than those of genes regulated by P_AOX1_ or P_GAP_ under specific culture conditions. These promoters were further transcriptionally characterized by reverse transcription-quantitative polymerase chain reaction (RT-qPCR) under different carbon sources, growth phases, and temperatures through the measurement of transcript levels of their corresponding genes, revealing distinct transcriptional profiles. Two major clusters were identified: one associated with genes upregulated during the stationary phase and the other with genes upregulated during the exponential phase. These transcriptional profiles provided a basis for the rational design of fed-batch strategies tailored to each promoter when driving heterologous gene expression. The *0208* and *HSP12* promoters showed particular potential at low specific growth rates, favoring high recombinant protein titers with reduced biomass accumulation. *In silico* analyses suggested that *0208* encodes a mitochondrial membrane-associated protein with a possible functional relationship to Hsp12. Overall, this work provides well-characterized *K. phaffii* strong promoter candidates, supporting future biotechnological applications.

## Introduction

Efficient transcription is essential for gene expression and a key determinant of heterologous protein production and metabolic engineering. Promoters regulate this process by providing specific DNA-binding sites for transcription factors (TFs) and the basal transcription machinery. For efficient gene expression, promoters should provide tight and finely tuned regulation while imposing minimal metabolic burden (Vogl & Glieder, 2013).

Over the past three decades, *Komagataella phaffii* (syn. *Pichia pastoris*) has become a widely used host for the production of heterologous proteins and metabolites. This yeast combines several advantageous features, including high cell density growth, efficient secretion, proper post-translational modifications, and high protein yields, that make it an attractive host for industrial applications (Khlebodarova et al., 2024). *K. phaffii* can grow on methanol as its sole carbon source and harbors strong, tightly regulated inducible promoters associated with the methanol utilization (*MUT*) pathway. Among them, the alcohol oxidase 1 promoter (P_AOX1_) is widely used due to its strong induction by methanol and repression by other carbon sources (Vogl & Glieder, 2013). This regulatory mechanism allows the decoupling of cell growth from heterologous protein production by switching from glycerol or glucose to methanol (Looser et al., 2015; Yang & Zhang, 2018). Other strong methanol-inducible promoters from genes within the *MUT* pathway, such as P_DAS_ (dihydroxyacetone synthase promoter) and P_FLD1_ (glutathione-dependent formaldehyde dehydrogenase 1 promoter), have also been investigated for regulating heterologous gene expression (Vogl & Glieder, 2013; Xu et al., 2018).

Despite its widespread use, P_AOX1_-based expression presents significant limitations due to its dependence on methanol, a hazardous substrate whose metabolism requires high oxygen levels and generates excess heat, all of which are factors that typically increase operational costs at an industrial scale (De Brabander et al., 2023; Bernat-Camps et al., 2023). To overcome these limitations, the use of methanol utilization slow (Mut^S^) strains has been proposed, since this approach reduces methanol consumption and improves feasibility for large-scale production (De Brabander et al., 2023; Viader-Salvadó et al., 2013a). Alternatively, methanol-independent expression strategies have also been developed, using constitutive promoters such as P_GAP_ (glyceraldehyde-3-phosphate dehydrogenase promoter) or other non-methanol-inducible promoters. These approaches offer safer and, in some cases, more cost-effective alternatives for industrial processes, leading to their increasing adoption in biotechnological applications (Yang & Zhang, 2018; Garrigós-Martínez et al., 2021; Bernat-Camps et al., 2023; Khlebodarova et al., 2024). P_GAP_-based systems enable high levels of constitutive expression and are well suited for fed-batch and continuous cultivation due to simpler bioprocess control and the absence of carbon source shifts. Moreover, they can achieve gram-per-liter protein titers comparable to those obtained with P_AOX1_-based systems, making them a viable alternative to the methanol-based P_AOX1_ strategies (Vogl & Glieder, 2013; Çalık et al., 2015; Yang & Zhang, 2018).

Although P_AOX1_ and P_GAP_ are widely used for methanol-inducible and constitutive expression, respectively, they are not suitable for all applications. High expression levels can hinder protein folding or impose a metabolic burden, and repeated use of a single promoter to co-express multiple genes in metabolic pathways can also lead to gene loss through recombination or transcription factor competition (Vogl & Glieder, 2013; Xu et al., 2018). While several alternative promoters have been identified in *K. phaffii*, their limited transcriptional strength or strict regulatory control restricts their applicability, highlighting the need for novel and versatile promoters (Xu et al., 2018).

RNA sequencing (RNA-seq) is a high-throughput technology that enables accurate transcriptome profiling by quantifying gene expression levels in a single assay. Advancements in next-generation sequencing (NGS) and the development of computational tools have improved the generation, analysis, and interpretation of transcriptomic data (Trapnell et al., 2012; Everaert et al., 2017). RNA-seq data has proven highly effective for identifying promoters in prokaryotes and yeast, including *K. phaffii*, where several studies have successfully used it to discover promoter candidates suitable for biotechnological applications (Xu et al., 2018; Dou et al., 2021; Zhang et al., 2022; Bernat-Camps et al., 2023).

In this work, we identified seven promoters from the most highly transcribed endogenous genes in carbon-limited *K. phaffii* cells as strong promoter candidates, using RNA-seq data from three recombinant strains grown with different carbon sources and under varied culture conditions. These promoters were further transcriptionally characterized using reverse transcription-quantitative PCR (RT-qPCR) in response to carbon sources (glucose and glycerol), cell growth phase, and temperature, through the measurement of transcript levels of their corresponding genes. Among the identified promoters, the *HSP12* and *0208* promoters corresponded to genes showing high transcript levels during the stationary phase. Because *0208* lacks a homolog in *Saccharomyces cerevisiae*, its transcriptional profile and the predicted protein structural model and membrane association were further analyzed in comparison with *HSP12*. Based on our results, we proposed cultivation strategies for recombinant protein production when these promoters are used to drive heterologous gene expression.

## Materials & Methods

### Strains

The *Komagataella phaffii* KM71 strains (KM71FTEII, KM71/P_GAP_-FTEII, KM71/P_GAP_-LAC412, and KM71PIC9) were previously constructed in our laboratory. The KM71FTEII (Viader-Salvadó et al., 2010) and KM71/P_GAP_-FTEII strains contain a beta-propeller phytase (FTEII) coding sequence (*FTEII* gene) under the control of the P_AOX1_ or P_GAP_ promoters, respectively, fused in-frame with the *S. cerevisiae* alpha-factor pre-pro-secretion signal coding sequence. The KM71/P_GAP_-LAC412 strain harbors the coding sequences for *β*-galactosidase (*LAC4* gene) and lactose permease (*LAC12* gene) from *Kluyveromyces lactis,* both under the control of P_GAP_. The KM71PIC9 strain contains the commercial vector pPIC9 (Thermo Fisher Scientific, Waltham, MA, USA) integrated into the yeast genome. In all cases, the *AOX1* transcriptional terminator (T_AOX1_) was used at the 3^′^ end of the expression cassette.

### RNA-seq experiments

Six cell samples from three different strains (KM71FTEII, KM71/P_GAP_-LAC412, and KM71PIC9) cultivated in five carbon sources under different culture conditions were analyzed by RNA-seq. KM71/P_GAP_-LAC412 and KM71PIC9 were grown in shake flasks using buffered minimal (BM) medium (100 mM phosphate buffer pH 6, 1.34% yeast nitrogen base (YNB), 4 × 10^−5^% biotin) supplemented with 30 mM glucose (BMGlc, 0.54% (w/v) glucose). KM71/P_GAP_-LAC412 was also grown in BM medium supplemented with 30 mM lactose (BMLac) or 30 mM lactose with 0.75% (v/v) methanol (BMLacMet). The incubations were performed at an initial optical density at 600 nm (OD_600_) of 0.1, at 30 °C, and 250 rpm. Cell samples were collected after 30 h of culture and are hereafter referred to as C1-Glc, C2-Glc, C3-Lac and C4-LacMet, respectively.

The KM71FTEII strain was grown in a 5 L BioFlo III bioreactor (New Brunswick Scientific Co. Inc.) using three steps (glycerol batch, glycerol-fed batch, and methanol-fed batch), as described previously (Viader-Salvadó et al., 2013b). The methanol-fed batch step was initiated when the biomass concentration reached 90 g dry cell weight (DCW)/L and was carried out at 24 °C, maintaining the methanol concentration in the broth at 1.5 g/L, and the pH at 6.0. Samples were harvested 45 min after the glycerol-fed batch was completed, just before the methanol-fed batch began (C5-Gly), and 47 h after the initiation of methanol induction (C6-Met).

Biological duplicate cultures were generated for samples C3-Lac and C6-Met, whereas the remaining transcriptomes were obtained from single biological cultures. No RNA-seq technical replicates were performed. Although the RNA-seq analysis was performed using three different strains, all three were derived from the *K. phaffii* KM71 host strain and differ mainly in the inserted expression cassette; therefore, they share the same genetic background. Sampling times were selected to capture the intended carbon-limited physiological state of each culture, including stationary phase, post-glycerol depletion, and methanol-limited induction during fed-batch cultivation.

### RNA sample preparations and RNA-seq data analyses

Cell samples were treated overnight with RNAlater solution (Ambion, Austin, TX, USA) and stored at −70 °C for no longer than one month prior to RNA extraction. Total RNA was isolated using the SV Total RNA Isolation System according to the manufacturer’s instructions for yeast (Promega, Madison, WI, USA). RNA integrity was assessed using the Agilent 2100 Bioanalyzer System (Agilent Technologies, Santa Clara, CA, USA) to ensure an RNA Integrity Number (RIN) score greater than or equal to 7. Total RNA concentrations ranged from 65 to 814 ng/µL, with RIN values between 7.8 and 9.8. cDNA libraries were constructed using the Illumina TruSeq RNA Sample Preparation kit (Illumina, San Diego, CA, USA). The eight TruSeq libraries generated had an average length of 420 bp and were paired-end sequenced (2 × 250 bp) on the Illumina MiSeq System at LANGEBIO-CINVESTAV (Irapuato, Gto, Mexico). Sixteen FASTQ files were obtained, two for each library, corresponding to the forward and reverse reads of each fragment in paired-end sequencing. The RNA-seq data were deposited in the NCBI Sequence Read Archive (SRA) under BioProject accession PRJNA930494. Individual BioSample and SRA run accession numbers for each transcriptome are provided in the Supplemental Information.

The *K. phaffii* GS115 genome sequence (Ref201010.fa; BOGAS), retrieved from ORCAE (http://bioinformatics.psb.ugent.be/orcae/, formerly known as BOGAS) (Sterck et al., 2012), was used as the reference. The genome sequence of each strain was customized by incorporating additional sequences into the reference genome, corresponding to the specific expression vector integrated into each strain’s genome.

All bioinformatics analyses were performed using the Galaxy online bioinformatics workflow management system (https://usegalaxy.org/), following the Tuxedo protocol, which includes the TopHat program and Cufflinks package (Trapnell et al., 2012). Low-quality reads (Phred < 20) were removed using FASTQ Quality Trimming v1.0.0 (Blankenberg et al., 2010). The remaining reads from the six RNA-seq datasets were independently aligned to each customized *K. phaffii* genome using TopHat v2.0.14. The resulting alignment files were processed using the Cufflinks v2.2.1 tool to generate assembled transcripts for each RNA-seq dataset, which were then identified using the Cuffcompare tool from the Cufflinks v2.2.1 package and each customized *K. phaffii* genome. The transcript levels of each identified transcript in each RNA-seq dataset were quantified as fragments per kilobase per million mapped reads (FPKM) using the Cufflinks v2.2.1 tool and used as proxies for the transcriptional activity of their corresponding genes. Total transcriptional activity (TTA) for each cell sample was estimated as the sum of the transcriptional activity values across all identified transcripts within each dataset.

RNA-seq data were analyzed using an exploratory and descriptive approach to identify highly transcribed genes within individual samples. Accordingly, no differential expression analysis was performed on the RNA-seq datasets.

### Identification of highly expressed genes by RNA-seq data analysis

The relative transcriptional activity (RTA) of the identified genes was calculated as the percentage of each gene’s transcriptional activity relative to the TTA in each transcriptome. Thus, RTA is mathematically proportional to transcripts per million (TPM) values (transcripts per million, RTA x 10^4^) and differs only by a constant scaling factor, since both metrics express transcript abundance relative to the sum of transcript abundances within the same sample. TPM has been proposed as a more consistent relative abundance measure than FPKM for comparisons among samples (Wagner, Kin & Lynch, 2012). RTA values of each transcriptome were represented in a box-and-whisker plot to identify genes with high outlier RTA values located above the third quartile (*i.e.,* RTA > third quartile + 1.5 times the interquartile range). Genes with an RTA value greater than 0.5% were selected from the set of genes with high outlier RTA values. A Venn diagram was then constructed using an online tool (https://bioinformatics.psb.ugent.be/webtools/Venn/) and the selected genes from the transcriptomes of C1-Glc to C5-Gly cell samples to identify common genes upregulated by methanol-independent promoters. A second Venn diagram was constructed to identify those genes with RTA value greater than 0.5% in the transcriptomes of cells grown in glucose- or glycerol-limited conditions (C1-Glc, C2-Glc, and C5-Gly). From this set of genes, those that exhibited RTA values higher than or comparable to the RTA of *FTEII* in the C6-Met transcriptome (strain KM71FTEII with the heterologous *FTEII* gene regulated by P_AOX1_), and/or the RTA of the endogenous glyceraldehyde-3-phosphate dehydrogenase (*GAPDH*) gene (regulated by P_GAP_) in the C1-Glc to C5-Gly transcriptomes were selected. Finally, genes containing an upstream inter-coding sequence (CDS) region greater than 1,300 bp and/or lacking a 5′-untranslated region (5′UTR) annotation in the *K. phaffii* GS115 genome were discarded.

### Transcriptome analysis validation by RT-qPCR

RT-qPCR was performed to validate the transcript levels of the selected upregulated genes in glucose- or glycerol-limited cultures identified by RNA-seq. Cell samples were obtained from three biological replicates of KM71/P_GAP_-LAC412 cells grown in glucose (C1-Glc) and KM71FTEII cells grown in glycerol (C5-Gly) under the same conditions used for RNA-seq analysis, and were stabilized overnight in RNAlater solution and stored at −70 °C for no longer than one month prior to RNA extraction. Total RNA preparations were obtained as described above for RNA-seq samples and treated with RQ1 RNase-free DNase (Promega Corporation, Madison, WI, USA) to remove genomic DNA. The RNA concentrations were within the range described above for the RNA-seq samples. Transcript amplifications were performed in two steps using 150 ng of total RNA from each sample, quantified by spectrophotometry at 260 nm with the NanoPhotometer Pearl (Implen GmbH, München, Germany). RNA purity was assessed by the A260/A280 ratio (1.9–2.1), and integrity was verified by agarose gel electrophoresis, showing intact rRNA bands and no evidence of degradation. Complementary DNA (cDNA) was synthesized using AffinityScript Multi-Temperature cDNA Synthesis Kit (Agilent Technologies, Santa Clara, CA, USA) and oligo(dT)_20_ in a final reaction volume of 20 µL, following the manufacturer’s instructions. The reaction included an initial denaturation step at 65 °C for 5 min, followed by incubation at 42 °C for 60 min and enzyme inactivation at 70 °C for 15 min.

Specific primer pairs were designed using Primer3 software (Untergasser et al., 2012) and the CDS sequences of the selected genes previously retrieved from the NCBI GenBank database (http://www.ncbi.nlm.nih.gov). All primers were designed within the coding sequence of each target gene. Primer specificity was verified using NCBI Primer-BLAST (https://www.ncbi.nlm.nih.gov/tools/primer-blast/) against the *K. phaffii* GS115 genome. Primer sequences, target accession numbers, and amplicon lengths are provided in Table S1. The qPCR reactions were performed in 20 *μ*L of reaction volume containing 0.3 µM of each primer, 2 µL of cDNA at an appropriate dilution according to the standard curve, and the Brilliant III Ultra-Fast SYBR Green QPCR Master Mix (Agilent Technologies, Santa Clara, CA, USA). All qPCR reactions were performed in biological triplicate and technical duplicate. A 40-cycle amplification program consisting of two steps (95 °C and 60 °C for 20 s each), preceded by an initial denaturation step at 95 °C for 3 min, was performed using the Mx3005P QPCR System (Agilent Technologies, Santa Clara, CA, USA). Cycle threshold (Ct) values were determined with MxPro-Mx3005P software v4.10 (Agilent Technologies) using a fixed fluorescence threshold of 2500 for all assays. No-template controls (NTCs) were included in every qPCR run, and no-RT controls confirmed the absence of genomic DNA (gDNA) amplification in all RNA samples. NTC reactions were considered acceptable only if no amplification signal was detected or if an amplification signal appeared at least five cycles later than the highest Ct value among samples within the same run. No outliers were detected among technical duplicates. Amplification data were exported to Microsoft Excel 365 (Microsoft Corporation) for further analysis. Melt curve analysis was performed at the end of each run for each amplicon to confirm amplification specificity. Calibration curves for each qPCR assay were generated from *K. phaffii* gDNA as a common standard, given that the targets are intronless and single copy. No splice variants are annotated for the selected target genes in *K. phaffii* GS115. All curves used the same gDNA stock (quantified by spectrophotometry at 260 nm), prepared as five-point 1:5 serial dilutions ranging from 25.00 to 0.04 ng (Table S2). All standard curves showed linearity (*r*^2^ ≥ 0.99). For each assay, PCR efficiency was derived from the slope of the linear regression of Ct *versus* the base-10 logarithm (log_10_) of gDNA amount. The lowest tested gDNA amount (0.04 ng) was consistently detected, and Ct variation between technical duplicates at this level ranged from 0.25 to 0.89 cycles across assays, reflecting intra-assay repeatability based on technical duplicates. Transcript levels for each gene of interest (GOI) were calculated relative to *VPS21* (PAS_chr4_0219) in the same sample by efficiency-corrected transformation of Ct values. Because both the GOI and *VPS21* were quantified using the same detection chemistry under identical conditions and amplification efficiencies were near 100%, the resulting values were numerically close to those obtained with the classical 2^−ΔCt^ approach, which is the recommended method for reporting normalized transcript levels of a GOI in individual samples (Schmittgen & Livak, 2008). The stability of *VPS21* was previously verified by assessing the variation (mean ± standard deviation, SD) in TPM values across the six transcriptomes. *VPS21* served as the endogenous normalizer in both RT-qPCR and RNA-seq analyses. All RT-qPCR assays were performed following published guidelines (Bustin et al., 2010).

### Characterization of native selected promoters

The selected promoters were transcriptionally characterized by RT-qPCR through the measurement of transcript levels of the genes regulated by their respective promoters in cells grown in two carbon sources at two temperatures. Batch cultures of the KM71/P_GAP_-FTEII strain were conducted in shake flasks in biological triplicate using BM medium supplemented with either 30 mM glucose (BMGlc, 0.54% (w/v) glucose) or 30 mM glycerol (BMGly, 0.28% (w/v) glycerol), both additionally supplemented with 0.1% (w/v) CaCl_2_. The cultures were incubated at either 24 °C or 30 °C resulting in four culture conditions: Glc24 (glucose, 24 °C), Glc30 (glucose, 30 °C), Gly24 (glycerol, 24 °C), and Gly30 (glycerol, 30 °C).

Samples from each culture were collected at 0, 3, 6, 12, 24, and 48 h to assess growth using a turbidimetric assay at OD_600_. Biomass concentrations were estimated based on the assumption that 1.0 OD_600_ unit corresponds to 0.23 g DCW/L (Herrera-Estala et al., 2022). Specific growth rates (μ) were determined from the slope of the natural log-linear regression of biomass concentration *versus* time during the exponential growth phase (3 to 12 h) and statistically compared using Student’s *t*-test with a significance level of 0.05. Growth kinetics data were also fitted using the nonlinear least-squares method to the integrated solution of the Verhulst-Pearl logistic equation (Tsoularis & Wallace, 2002), implemented in a custom Python program utilizing the *curve_fit* function from the SciPy open-source library (https://scipy.org/).

Cell samples were harvested by centrifugation (16,000 × g, 10 min, 4 °C) at 6 and 12 h during the exponential growth phase, and at 24 and 48 h during the stationary phase. RT-qPCR assays were performed as described above, using the same specific primers and SYBR Green as the intercalating agent. PrimeTime qPCR Probe Assays (Integrated DNA Technologies, Inc.) were used for the *FTEII* and *GAPDH* gene analyses as previously described (Herrera-Estala et al., 2022). Each assay included a target-specific primer pair and a hydrolysis probe labeled at the 5′ end with FAM and at the 3′ end with ZEN/Iowa Black FQ. Sampling times and temperatures were selected to evaluate transcriptional behavior during exponential and stationary growth phases under standard (30 °C) and reduced-temperature cultivation conditions.

Transcript levels of each gene from cells grown under the four evaluated culture conditions were calculated as fold changes relative to *VPS21* in the same sample and statistically compared using a Student’s *t*-test with a significance level of 0.05. Statistical analyses were performed using Microsoft Excel 365 (Microsoft Corporation). Moreover, transcript level data were normalized to the maximum transcript level for each gene and a principal component analysis (PCA) was applied to the normalized data to reduce dimensionality. The PCA was performed using a custom Python script, with the *pca.fit_transform* function from the scikit-learn library (Pedregosa et al., 2011). The first two principal components (PC1 and PC2) were considered together as explaining most of the variance in the data. Clustering patterns among transcript levels, related to the carbon source, cell growth phase, and temperature were observed based on the distribution of genes in the space defined by PC1 and PC2. To confirm the clusters visualized in the PCA, a hierarchical clustering analysis was also applied to the normalized transcript level data. The clustering was performed using Ward’s algorithm in a custom Python script with the *linkage* function from the SciPy library (https://scipy.org/).

### Bioinformatics analysis of the upstream regions and the encoded amino acid sequences of the *K. phaffii HSP12* and *0208* genes

An *in silico* analysis of the upstream inter-CDS region of the *HSP12* (PAS_chr4_0627) and *0208* (PAS_chr2-2_0208) genes from the *K. phaffii* GS115 genome was conducted to predict putative transcription factor-binding sites (TFBS) for Msn2/Msn4 and Hsf1, using the FIMO tool (Grant, Bailey & Noble, 2011) and JASPAR data for fungi (Rauluseviciute et al., 2024). The percentage of sequence identity between Hsp12 and 0208 amino acid sequences was calculated using the global Needleman-Wunsch pairwise alignment implemented in the BLAST tools (https://blast.ncbi.nlm.nih.gov/). Molecular models of the Hsp12 and 0208 proteins were retrieved from the AlphaFold Protein Structure Database (Varadi et al., 2024) and visualized with UCSF Chimera software (Pettersen et al., 2004). Helical wheel representations of the alpha-helices in the Hsp12 and 0208 structures were generated using the Wang and Saladi online tool (https://clemlab.github.io/helicalwheel/). The subcellular localization and membrane association type of the proteins Hsp12 and 0208 were predicted using DeepLoc 2.1 (Odum et al., 2024).

## Results

### RNA-seq dataset analysis

The number of reads for each RNA-seq dataset ranged from 2,483,548 to 5,994,874, corresponding to an estimated sequencing depth of approximately 80× to 200× based on the total annotated exonic length of the *K. phaffii* GS115 reference genome (7.5 Mb), which provided sufficient data for further analysis (Fonseca, Marioni & Brazma, 2014).

The percentage of reads lost after trimming ranged from 0.007% to 0.038% for both the 5′ and 3′ reads, indicating high read quality and efficient trimming. The number of reads mapped to the customized *K. phaffii* genome using the TopHat tool was similar at both the 5′ end (82–95%) and the 3′ end (84–94%), which is comparable to other methods that typically range from 79% to 92% (Kim et al., 2013). Cuffcompare analysis identified 5,203 transcripts across the six transcriptomes, representing 98% of the 5,313 genes annotated for the *K. phaffii* GS115 strain (De Schutter et al., 2009). The number of identified transcripts varied among strains and culture conditions. The KM71PIC9 and KM71FTEII strains showed 5.7% and 3.0% fewer transcripts, respectively, compared to the average number of identified transcripts across the three KM71/P_GAP_-LAC412 transcriptomes (Table 1). The KM71PIC9 strain in the stationary phase after growing in glucose (C2-Glc) exhibited the highest TTA value among the six RNA-seq datasets, while the KM71FTEII methanol-grown cells (C6-Met) showed the lowest TTA value. The KM71PIC9 strain displayed TTA values 1.4 to 1.6-fold higher than those of the KM71/P_GAP_-LAC412 strain. These values were also 2.0- to 2.2-fold higher than those of the KM71FTEII strain (Table 1).

**Table 1:** Total transcriptional activity (TTA) in FPKM (fragments per kilobase per million mapped reads), number of identified transcripts (IT), and percentage of IT relative to the 5,313 genes annotated for the *K. phaffii* GS115 strain (De Schutter et al., 2009) in the six RNA-seq datasets.

**Strain**	**RNA-seq dataset**	**TTA**	**Number of IT**	**Percentage of IT**
KM71PIC9	C2-Glc	1940,043	4,713	89
KM71/P_GAP_-LAC412	C1-Glc	1381,429	4,963	93
	C3-Lac	1388,702	5,038	95
	C4-LacMet	1216,447	4,997	94
KM71FTEII	C5-Gly	978,304	4,836	91
	C6-Met	899,638	4,858	91

### Identification of highly expressed genes by RNA-seq data analysis

**Figure 1:**
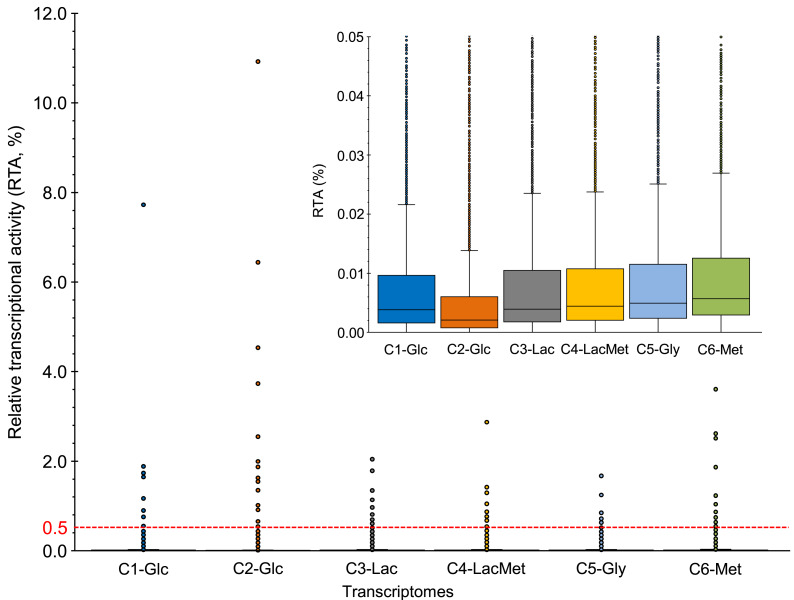
Box-and-whisker plot showing the relative transcriptional activity (RTA) of the identified genes across six transcriptomes. The RNA-seq dataset corresponds to cells grown in glucose (C1-Glc and C2-Glc), lactose (C3-Lac), a lactose-methanol mixture (C4-LacMet), glycerol (C5-Gly), and methanol (C6-Met). Dots represent genes with high outlier RTA values above the third quartile. The red dashed line indicates the RTA threshold of 0.5%. The inset shows a zoomed view of the lower RTA range to allow visualization of the boxplot distributions.

The boxplot analyses (Fig. 1) showed that the number of genes with high outlier RTA values ranged from 559 to 663 across the six transcriptomes, representing an average of 13% relative to the number of identified transcripts. A total of 78 different genes with an RTA greater than 0.5% were identified across all transcriptomes. The number of these genes per transcriptome ranged from 24 to 31, representing an average of 0.53% relative to the number of identified transcripts.

**Figure 2:**
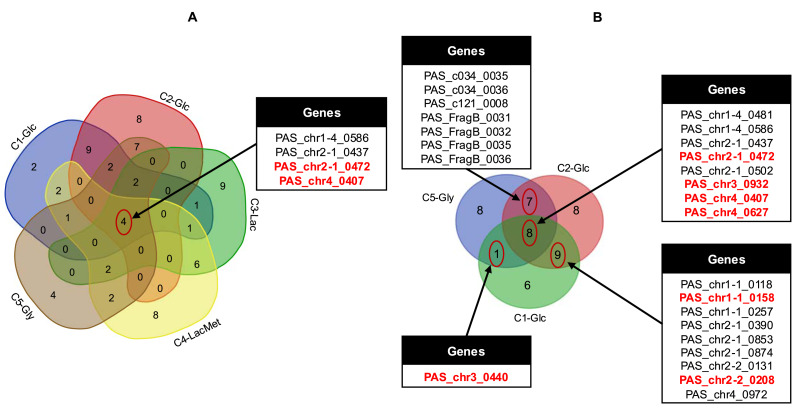
Venn diagrams showing the number of common genes across transcriptomes with RTA > 0.5%. (A) Cells grown in glucose (C1-Glc and C2-Glc), lactose (C3-Lac), a lactose-methanol mixture (C4-LacMet), and glycerol (C5-Gly). (B) Cells grown in glucose (C1-Glc and C2-Glc) and glycerol (C5-Gly). The seven selected genes are highlighted in bold red.

The Venn diagram constructed with 70 genes (RTA > 0.5%) from the C1-Glc to C5-Gly transcriptomes (Fig. 2A) revealed four shared genes. These genes were highly transcribed under the control of promoters that are not induced by methanol. The second Venn diagram (Fig. 2B), constructed to identify common genes highly transcribed in glucose- or glycerol-limited conditions, showed 25 shared genes among the 47 different genes with an RTA > 0.5% in the C1-Glc, C2-Glc, and C5-Gly transcriptomes. From this subset of 25, genes lacking a 5′UTR annotation or with upstream inter-CDS regions longer than 1,300 bp were discarded, resulting in the identification of seven highly transcribed genes in glucose- or glycerol-limited cultures (Table 2). For convenience, these seven genes were hereinafter referred to by the corresponding *S. cerevisiae* homologous gene names, except for PAS_chr2-2_0208, which was referred to as the *0208* gene (Table 2).

**Table 2:** Highly transcribed genes identified in cells grown under glucose- or glycerol-limited conditions.

**Gene ID** ^a^	**Gene name** ^b^	**Protein**	**CDS (bp)**	**Inter-CDS (bp)**
PAS_chr4_0627	*HSP12*	Heat shock protein 12	354	666
PAS_chr2-2_0208	*0208*	Hypothetical structural protein	507	399
PAS_chr3_0932	*FDH1*	NAD^+^-dependent formate dehydrogenase	1,098	1,131
PAS_chr3_0440	*JEN1*	Monocarboxylate permeases	1,659	909
PAS_chr1-1_0158	*ADY2*	Monocarboxylate permeases	792	1,039
PAS_chr2-1_0472	*ADH2*	Mitochondrial alcohol dehydrogenase	1,053	1,214
PAS_chr4_0407	*TMA10*	Unknown protein associated with ribosomes	312	840

**Notes.**

a Based on the annotation of the *K. phaffii* GS115 strain (De Schutter et al., 2009).

b Based on the corresponding *S. cerevisiae* homologous gene, except for PAS_chr2-2_0208, which was referred to as the *0208* gene.

CDS, coding sequence.

**Figure 3:**
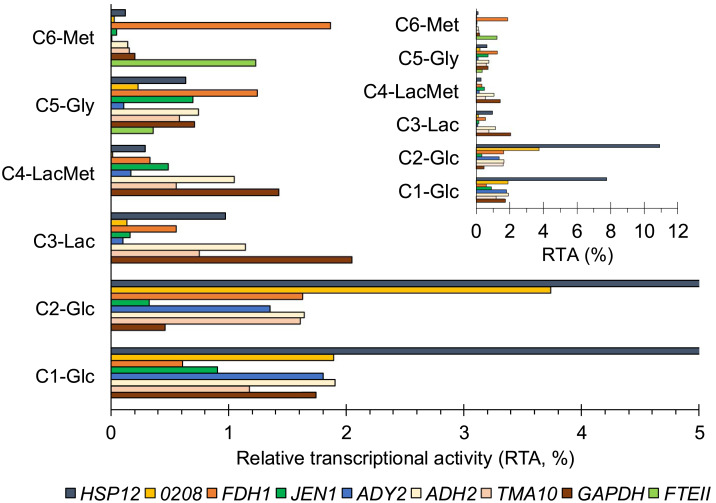
Relative transcriptional activity (RTA) of *HSP12*, *0208*, *FDH1*, *JEN1*, *ADY2*, *ADH2*, and *TMA10* compared with *GAPDH* and *FTEII* across transcriptomes. Cells grown in glucose (C1-Glc, C2-Glc), lactose (C3-Lac), a lactose–methanol mixture (C4-LacMet), glycerol (C5-Gly), and methanol (C6-Met). The inset shows the same data using the full RTA scale.

Figure 3 shows the RTA values of the seven selected genes for the six analyzed transcriptomes. The RTA of the heterologous *FTEII* gene in the C6-Met transcriptome, along with the RTA of the *GAPDH* gene in the C1-Glc transcriptome, served as references for comparing the RTA values of the selected genes. Although the RNA-seq datasets were obtained from strains sharing the same KM71 genetic background and from samples collected under carbon-limited physiological states, the culture conditions differed in carbon source, sampling time, and growth phase. Therefore, the datasets were used primarily to identify the most highly transcribed genes within each condition-specific transcriptome, and cross-condition comparisons of RTA values were interpreted cautiously as relative transcript abundance rather than as absolute transcript amounts.

As expected, the RTA value of the *FTEII* gene was 6.0-fold higher than that of the *GAPDH* gene in KM71FTEII cells when methanol was used as the carbon source (C6-Met transcriptome). Moreover, the RTA value of the *GAPDH* gene was twice as high as that of the *FTEII* gene under glycerol-limited conditions (C5-Gly transcriptome), since in this strain *GAPDH* is regulated by P_GAP_, whereas *FTEII* is under the control of P_AOX1_. The highest RTA values for the *GAPDH* gene were observed in the KM71/P_GAP_-LAC412 strain under glucose- and lactose-limited conditions (C1-Glc and C3-Lac). The genes *HSP12*, *0208*, *FDH1*, *ADY2*, *ADH2* (previously known as *ADH3*), and *TMA10* showed RTA values equal to or higher than those of *GAPDH* in at least one strain (KM71/P_GAP_-LAC412 or KM71PIC9) under glucose-limited conditions (C1-Glc and C2-Glc). Furthermore, these RTA values were also higher than that of *FTEII* in KM71FTEII cells grown on glycerol or methanol (C5-Gly or C6-Met). Similarly, in the KM71FTEII strain under glycerol-limited conditions (C5-Gly), the genes *HSP12*, *FDH1*, *JEN1*, *ADH2* and *TMA10* exhibited RTA values equal to or higher than those of both *GAPDH* and *FTEII*, except for *TMA10*, whose RTA value remained below that of *GAPDH*. Only the *FDH1* gene had an RTA value higher than that of the heterologous *FTEII* gene in the KM71FTEII strain in the presence of methanol as the carbon source (C6-Met). The highest RTA values were observed for the *HSP12* gene in the KM71/P_GAP_-LAC412 and KM71PIC9 strains under glucose-limited conditions (C1-Glc and C2-Glc), with values 4.5- and 23.7-fold higher than those of the *GAPDH* gene, respectively. Another outstanding RTA value was recorded for the *0208* gene in the KM71PIC9 strain under glucose-limited conditions (C2-Glc transcriptome), with a value 8.1-fold higher than that of the *GAPDH* gene. These results demonstrate that the identified genes showed RTA values equal to or higher than those of the *GAPDH* or *FTEII* genes under glucose- or glycerol-limited conditions, meeting the specified criteria.

### Validation of the RNA-seq analysis by RT-qPCR

The transcript levels of *VPS21* were used to normalize those of the selected genes, as *VPS21* exhibited consistent TPM values across all the RNA-seq datasets, with an average of 228 ± 54 (mean ± SD).

**Figure 4:**
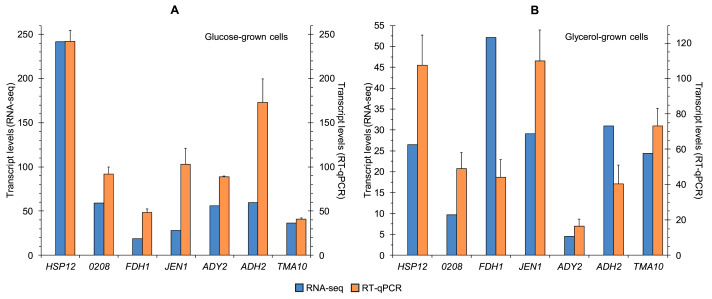
Comparison of transcript levels by RNA-seq and RT-qPCR. Transcript levels of *HSP12*, *0208*, *FDH1*, *JEN1*, *ADY2*, *ADH2*, and *TMA10* as determined by RNA-seq and RT-qPCR, calculated as fold changes relative to *VPS21*. (A) Glucose-grown KM71/P_GAP_-LAC412 cells (C1-Glc). (B) glycerol-grown KM71FTEII cells (C5-Gly). RT-qPCR data are mean ± standard error from three independent cultures.

Transcript levels of the seven selected genes, determined by RNA-seq and RT-qPCR and expressed as fold changes relative to *VPS21*, are shown in Figs. 4A and 4B. Panels correspond to glucose-grown KM71/P_GAP_-LAC412 cells (C1-Glc) and glycerol-grown KM71FTEII cells (C5-Gly), respectively. Both RNA-seq and RT-qPCR revealed a similar transcriptional profile for glucose-grown KM71/P_GAP_-LAC412 cells. A linear correlation was observed between the two techniques (*R*^2^ = 0.91) when *ADH2* was excluded from the analysis. For glycerol-grown KM71FTEII cells, the two techniques also correlated linearly (*R*^2^ = 0.92) when *FDH1* and *ADH2* were excluded. Under glucose conditions, RT-qPCR consistently detected higher transcript levels than RNA-seq across most genes. Notable differences were observed for *ADH2* and *JEN1*, which showed an average RT-qPCR value 3.6-fold higher than RNA-seq. However, when considering all genes, RT-qPCR values were, on average, 2.3-fold higher than those obtained by RNA-seq. This pattern was not observed in glycerol-grown cells, where RT-qPCR values were, on average, 7-fold higher than RNA-seq for all genes, except for *ADH2* and *FDH1*, which differed by 2-fold.

Overall, RT-qPCR results were consistent with RNA-seq data, supporting the validity of the RNA-seq analysis for identifying and selecting genes of interest for further evaluation.

### Characterization of native selected promoters

The growth kinetics of the KM71/P_GAP_-FTEII strain cultivated in glucose or glycerol as carbon sources at 24 °C or 30 °C (Fig. S1) showed a characteristic sigmoidal growth pattern. Biomass concentrations followed an exponential trend from 3 to 12 h of culture, with μ values ranging from 0.230 ± 0.021 to 0.262 ± 0.010 h^−1^, reaching a plateau after 24 h in all four kinetic assays. A higher maximum biomass concentration was achieved in glucose-grown cultures compared to those using glycerol. No significant differences in *μ* values were observed between the carbon sources and culture temperatures tested.

**Figure 5:**
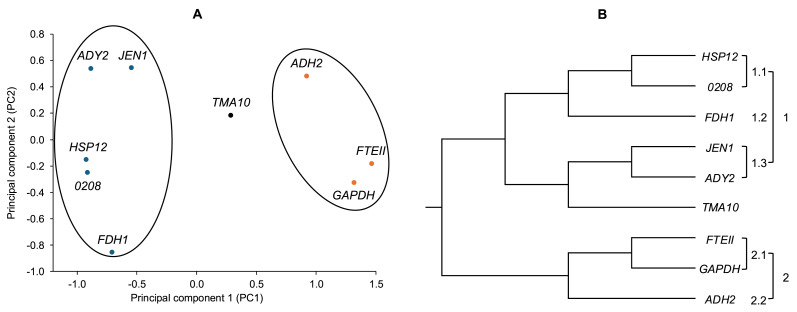
Principal component analysis (PCA) and hierarchical clustering of gene transcript levels in cells grown under different conditions. (A) PCA and (B) cluster analysis of transcript level data for *HSP12*, *0208*, *FDH1*, *JEN1*, *ADY2*, *ADH2*, *TMA10*, and the reference genes *GAPDH* and *FTEII* from KM71/P_GAP_-FTEII cells grown in glucose or glycerol at 24 °C or 30 °C and sampled at 6, 12, 24, and 48 h. Transcript levels were normalized to the highest value for each gene. In the PCA plot, the two gene clusters are enclosed within ellipses. In the dendrogram, clusters and subclusters are indicated by numbers.

The transcript levels of the selected genes and the two reference genes (*GAPDH* and *FTEII*) in the KM71/P_GAP_-FTEII strain varied with the carbon source, cell growth phase, and temperature. The PCA (Fig. 5A) and the clustering dendrogram (Fig. 5B) revealed two well-defined gene clusters: (1) *HSP12*, *0208*, *FDH1*, *JEN1*, and *ADY2*; and (2) *GAPDH*, *FTEII*, and *ADH2*. PC1 explained 66.0% of the variance and PC2 explained 14.8%, together accounting for 80.8% of the data variance. The main factor influencing PC1 was the growth phase, which separated the gene set into two clusters. Genes in cluster 1 were upregulated in the stationary phase, while genes in cluster 2 were upregulated in the exponential growth phase. However, each gene exhibited a distinct transcript level pattern with unique characteristics that defined subclusters. *TMA10* showed intermediate behavior between the two clusters. PCA grouped it closer to cluster 2, whereas the clustering analysis assigned it to cluster 1. Euclidean distances from Ward’s algorithm placed *TMA10* closest to *JEN1*, supporting its inclusion in cluster 1. Nevertheless, the distances to *ADH2* and *GAPDH* were the second and third shortest, respectively, suggesting a possible association with cluster 2. *ADY2*, a cluster 1 gene, was the fourth closest to *TMA10*. Both the PCA and clustering analysis also showed the presence of three subclusters within cluster 1: 1.1 (*HSP12* and *0208*), 1.2 (*FDH1*), and 1.3 (*JEN1* and *ADY2*). Similarly, cluster 2 is composed of two subclusters: 2.1 (*FTEII* and *GAPDH*) and 2.2 (*ADH2*).

**Figure 6:**
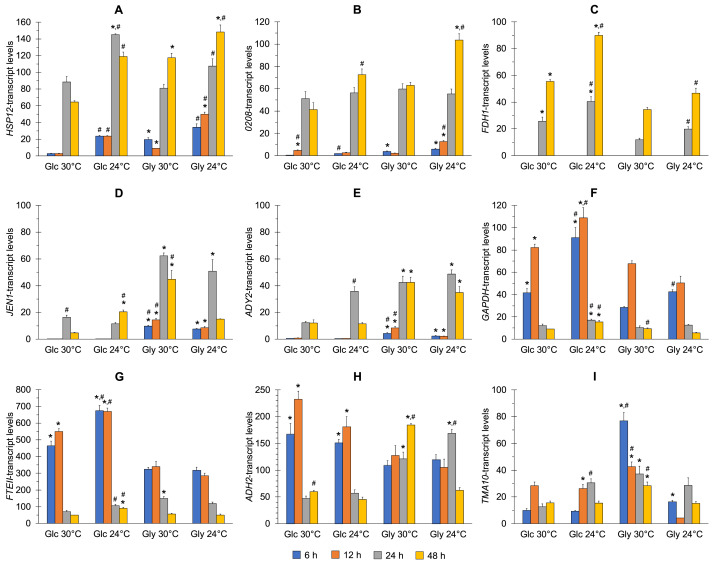
Time-course transcriptional profiles of nine genes in KM71/P_GAP_-FTEII cells grown in glucose or glycerol at 24 °C or 30 °C. (A) *HSP12*, (B) *0208*, (C) *FDH1*, (D) *JEN1*, (E) *ADY2*, (F) *GAPDH*, (G) *FTEII*, (H) *ADH2*, and (I) *TMA10*. Transcriptional profiles are shown as transcript levels (fold change relative to *VPS21*). Data are the mean ± standard error from three independent cultures. Symbols indicate significant differences (*p* < 0.05): (*) between cell samples grown in different carbon sources at the same temperature, and (#) between cell samples grown at different temperatures with the same carbon source. The symbols are placed above the higher values in each pairwise comparison.

Figure 6 shows the transcript levels of the evaluated genes in KM71/P_GAP_-FTEII cells grown in glucose or glycerol at 24 °C or 30 °C. The statistical details of the pairwise comparisons are provided in the Supplementary Material: Table S3 summarizes the effects of the carbon source, and Table S4 the effects of temperature. In both tables, the conditions associated with increased transcript levels are also indicated.

The *HSP12* and *0208* genes (subcluster 1.1) showed similar transcriptional patterns, tending to be upregulated in glycerol relative to glucose cultures and at 24 °C compared to 30 °C (Figs. 6A and 6B). For *HSP12*, transcript levels did not differ between carbon sources at 6 h/24 °C or at 24 h/30 °C. However, transcript levels were higher in glycerol-grown cells at 12 and 48 h/24 °C and at 6, 12, and 48 h/30 °C compared to glucose. Transcript levels were more abundant in glucose than in glycerol cultures only at 24 h/24 °C. *HSP12* showed higher transcript levels at 24 °C than at 30 °C in both glucose- and glycerol-grown cells at the two growth phases. Peak transcript levels occurred at 24 °C/24 h in glucose and 24 °C/48 h in glycerol, reaching values 1.4-fold higher than the maximum detected for the *0208* and *GAPDH* genes. *0208* showed increased transcript levels in glycerol-grown cells than in glucose-grown cells at 24 °C during most culture times, and at 30 °C only at 6 h. Transcript levels were also higher at 24 °C than at 30 °C in glucose (6 and 48 h) and glycerol (12 and 48 h) cultures. The maximum transcript levels were detected at 48 h in glycerol at 24 °C, comparable to those of *GAPDH* at 12 h in glucose at 24 °C.

The *FDH1* gene (subcluster 1.2) exhibited higher transcript levels in glucose- than in glycerol-grown cells during the stationary phase at both 24 °C and 30 °C, with higher transcript levels at 24 °C in both carbon sources (Fig. 6C). Its peak transcript levels occurred in glucose cultures at 24 °C and 48 h, reaching values 1.2-fold lower than those of *GAPDH*.

*JEN1* and *ADY2* (subcluster 1.3) exhibited similar transcriptional patterns, with higher transcript levels in glycerol- than in glucose-grown cells across most culture times and temperatures (Figs. 6D and 6E). *JEN1* transcript levels were higher at 24 °C than at 30 °C in glucose cultures at 48 h, but the opposite was observed at 24 h. In glycerol cultures, *JEN1* consistently showed higher transcript levels at 30 °C than at 24 °C throughout all growth phases. *ADY2* transcript levels were higher at 24 °C than at 30 °C at 24 h in glucose-grown cells, whereas in glycerol-grown cells, they were higher at 30 °C at 6 and 12 h. *ADY2* reached its maximum transcript levels during the stationary phase in glycerol-grown cells at both temperatures. In contrast, *JEN1* transcript levels peaked at 30 °C in glycerol during the stationary phase, reaching values 1.3-fold higher than *ADY2* and 1.7-fold lower than *GAPDH*.

Despite differences in absolute values, *GAPDH* and *FTEII* (subcluster 2.1) exhibited similar transcript level patterns across the tested conditions, showing consistent regulation in response to carbon source and temperature (Figs. 6F and 6G). *GAPDH* displayed elevated transcript levels in glucose- compared to glycerol-grown cells at 24 °C during both growth phases, and at 30 °C only during the exponential phase. Its transcript levels were higher at 24 °C than at 30 °C in glucose during both growth phases and in glycerol at 6 h. Maximum transcript levels were observed in glucose cultures at 12 h/24 °C. *FTEII* exhibited higher transcript levels in glucose- than in glycerol-grown cells at both 24 °C and 30 °C during the exponential phase and at 48 h/24 °C. In contrast, glycerol-grown cells had higher *FTEII* transcript levels than glucose-grown cells at 24 h/30 °C. Its transcript levels were also higher at 24 °C than at 30 °C in glucose cultures during both growth phases, while no significant temperature-related differences were observed in glycerol cultures. The highest *FTEII* transcript levels occurred in glucose at 6 and 12 h/24 °C, reaching values 6.2-fold above *GAPDH* maximum transcript levels.

The *ADH2* gene (subcluster 2.2) showed higher transcript levels in glucose- than in glycerol-grown cells during the exponential phase at both temperatures, while the opposite was observed during the stationary phase, except at 48 h/24 °C (Fig. 6H). No significant temperature-related differences were found in glucose cultures, whereas in glycerol cultures, transcript levels were higher at 24 h at 24 °C than at 30 °C, but the opposite was observed at 48 h. Its maximum transcript levels were detected in glucose at 12 h at both temperatures, reaching values 2.1-fold higher than *GAPDH*.

The *TMA10* gene (located between the two gene clusters) exhibited higher transcript levels in glycerol- than in glucose-grown cells at 30 °C during both growth phases, while at 24 °C this pattern was observed only at 6 h (Fig. 6I). *TMA10* transcript levels were higher in glucose- than glycerol-grown cells at 12 h/24 °C. In glycerol, transcript levels were higher at 30 °C than at 24 °C, except at 24 h. In glucose, higher levels at 24 °C were only observed at 24 h. *TMA10* reached its maximum transcript levels in glycerol at 30 °C and 6 h, being 1.4-fold lower than the *GAPDH* peak.

### Bioinformatics analysis of the upstream inter-CDS regions and the encoded amino acid sequences of the *K. phaffii HSP12* and *0208* genes

*In silico* analysis of the upstream inter-CDS region of *HSP12* identified 6 putative TFBS for Msn2/Msn4 and 10 for Hsf1 (Table S5). In the corresponding region of *0208*, 2 and 4 putative sites were identified for the same TFs, respectively (Table S6). A schematic representation of the positions of these predicted TFBS is provided in the Supplementary Material, below Tables S5 and S6. The Hsp12 and 0208 proteins shared only 16% sequence identity. Molecular models for Hsp12 and 0208 proteins revealed a similar fold (Fig. S2), with four alpha-helices covering 73% and 70% of the protein, respectively. Nevertheless, in Hsp12, the largest helix (41 residues) corresponds to H4, whereas in 0208, the largest helix (40 residues) corresponds to H1. The helical wheel representation for the two protein structures (Fig. S2) showed hydrophobic residues clustered mainly on one side of each helix, with polar and charged residues on the opposite side. Subcellular localization and membrane association analyses indicated that Hsp12 and 0208 are soluble proteins associated with membranes but not integrated as transmembrane proteins (Table S7). Hsp12 may interact peripherally with the plasma membrane, whereas 0208 is more likely to associate with the mitochondrial outer membrane (Table S8).

## Discussion

Transcription is the first step in gene expression, and its activity is primarily determined by the promoter. Although several functional regulatory elements have been identified in *K. phaffii* beyond P_AOX1_ and P_GAP_ (Yang & Zhang, 2018; Xu et al., 2018; Dou et al., 2021; Zhang et al., 2022), the currently available promoters remain limited, highlighting the need for continued screening and discovery of novel ones. In this study, we used RNA-seq to identify native promoters as alternatives to conventional ones, focusing on genes with high transcript levels under different carbon sources and culture conditions.

The identification of an average of 5,203 transcripts, representing 98% of annotated genes in the *K. phaffii* GS115 strain, is consistent with previous RNA-seq studies (Yu et al., 2017; Dou et al., 2021), supporting the reliability of the experimental protocols used. Variations in FPKM-based TTA and in the number of identified transcripts among strains and conditions suggest strain-specific transcriptional responses and adaptations to culture environments. Comparable transcript counts (4,410–4,897) during the stationary phase in batch cultures of the GS115 strain grown on glucose, glycerol, or methanol have been reported (Dou et al., 2021). The relatively lower transcript counts observed in the KM71PIC9 and KM71FTEII strains compared to the other strains may reflect differences in global transcriptional output under the evaluated conditions.

The metabolic response of each strain influenced both TTA and individual gene transcriptional activity depending on the strain and carbon source. Unlike previous studies that used raw FPKM values to identify highly expressed genes (Xu et al., 2018; Yu et al., 2017; Dou et al., 2021), we normalized FPKM to TTA to obtain RTA values, enabling box plot analysis to detect the most highly transcribed genes. Genes with outlier RTA values > 0.5% represented only 0.53% of the average transcripts identified per transcriptome. Our selection criterion for the identification of strong promoter candidates (RTA > 0.5%) corresponds to transcript levels at least 24-fold higher than the transcriptome-wide mean. This difference substantially exceeds the variability attributed individually to transcriptional terminators in *K. phaffii*, approximately 5-fold (Ito et al., 2020), and to locus-dependent effects in single-copy constructs in *K. phaffii*, about 3-fold (Brady et al., 2020). While contributions from CDS features in endogenous loci cannot be fully excluded, these bounds suggest that the observed extreme RTA for the genes regulated by the selected promoters is unlikely to be explained by terminator or locus effects alone. Therefore, these promoters may be considered strong promoter candidates, although intrinsic promoter strength should be established using reporter gene constructs. We did not attempt to define the exact minimal functional regulatory unit of the seven selected promoters. Instead, promoters from genes with annotated 5′ UTRs and upstream inter-CDS regions shorter than 1,300 bp were prioritized, so that the complete inter-CDS regions could be readily used as an initial promoter fragment in future expression constructs. Further promoter truncation analysis and functional validation will be necessary to define the minimal functional regulatory unit of these promoters.

Among the 70 genes with RTA > 0.5% identified across the five transcriptomes from three phenotypically distinct *K. phaffii* strains, only four were consistently detected in all datasets. Seventeen genes (0.35% of total transcripts) were commonly expressed in transcriptomes from cells grown in glucose- or glycerol-limited conditions (C1-Glc, C2-Glc and C5-Gly), of which seven were selected. These genes were categorized in two groups: those with high transcript levels under glucose-limited conditions (C1-Glc or C2-Glc) and those with high transcript levels under glycerol-limited conditions (C5-Gly). Among all candidates, *HSP12* and *0208* exhibited the highest RTA values compared to *GAPDH* under glucose-limited conditions, supporting the potential of their promoters as strong promoter candidates. These findings highlight the utility of combining RNA-seq data and bioinformatics tools with RTA-based analyses, box plots, and Venn diagrams to identify a subset of endogenous promoters as strong promoter candidates.

Currently, RNA-seq is widely recognized as a reference method for transcriptome-wide gene expression quantification. In this study, the transcript levels of seven genes identified from RNA-seq data were validated by RT-qPCR. Both methods showed a similar transcriptional pattern for these genes. However, overall, RT-qPCR consistently detected higher transcript levels than RNA-seq across most genes. RNA-seq has been validated as a reliable tool for transcriptome-wide gene expression quantification due to its high correlation with RT-qPCR measurements. However, RT-qPCR is still recommended for validating transcript levels of genes with low expression levels or complex transcript structures (Everaert et al., 2017). Given the variability in gene expression across experimental conditions, the context-specific selection of reference genes is essential. Therefore, RNA-seq is being progressively used to identify suitable reference genes for accurate RT-qPCR normalization (Liang et al., 2020). Traditional housekeeping genes may not remain stable under all conditions. Therefore, computational tools are increasingly used to identify suitable reference genes directly from RNA-seq data, ensuring reliable normalization and accurate quantification (De Brito et al., 2024). Based on its consistent RTA values across all transcriptomes, we identified *VPS21* as a stable reference gene from the RNA-seq data and used it to normalize RT-qPCR data for both the validation of the RNA-seq results and the characterization of transcript levels of the selected genes. Given its stable expression, the use of a single reference gene was considered sufficient for normalization. RT-qPCR validation of RNA-seq data confirmed the reliability of RNA-seq for transcriptome-wide gene expression quantification. Overall, RNA-seq and RT-qPCR showed strong concordance (*R*^2^ > 0.9 after excluding two influential outliers). The exclusion of *ADH2* (from glucose-grown cells) and *ADH2*/*FDH1* (from glycerol-grown cells) reflects gene-specific discrepancies that have been previously reported when comparing both techniques, even though global agreement is typically high (Everaert et al., 2017). These deviations are consistent with technique-specific limitations rather than biological inconsistencies, thereby supporting the robustness of the validation. The trend of higher transcript levels detected by RT-qPCR compared to RNA-seq is also consistent with previous reports, emphasizing the need for careful interpretation of RNA-seq data (Everaert et al., 2017; Liang et al., 2020).

Although most RNA-seq data were generated from single biological cultures, their high technical reproducibility (Marioni et al., 2008) supports their use for transcriptome-wide screening. In this study, RNA-seq was used to identify a subset of endogenous promoters from the most highly transcribed *K. phaffii* genes as strong promoter candidates. Their transcriptional characterization under different carbon sources, growth phases, and temperatures was subsequently conducted by RT-qPCR using biological replicates, ensuring that conclusions regarding these strong promoter candidates are based on biologically validated data.

The classification of the selected genes into two clusters was primarily influenced by the regulation associated with the cell growth phase (*i.e.,* cluster 1: genes upregulated in the stationary phase, cluster 2: genes upregulated in the exponential growth phase).

The *in silico* analyses of the *HSP12* and *0208* upstream regions and encoded proteins were included to gain additional insight into *0208*, which was the only selected gene encoding a hypothetical protein of unknown function. Because *0208* showed a transcriptional pattern similar to that of *HSP12* and both genes were classified into the same subcluster (1.1), *HSP12* was used as a biologically relevant reference for comparative analysis. In *S. cerevisiae*, *HSP12* encodes the plasma membrane-associated heat shock protein 12, which stabilizes this membrane by modulating its fluidity (Welker et al., 2010). In *K. phaffii*, Hsp12 has been linked to the repair of stress-induced cell damage, facilitating adaptation to environmental stresses (Wang et al., 2021). This is consistent with our observation that its transcript levels increased during the stationary phase, where several environmental stresses arise due to high cell density and carbon source depletion. The observed *HSP12* downregulation during the exponential phase and upregulation in the stationary phase also aligns with previous reports in *K. phaffii* (Zhang et al., 2022; Dou et al., 2021). Thus, the *HSP12* promoter should be interpreted as a stress-responsive and condition-dependent promoter candidate. The amphipathic properties of the Hsp12 alpha-helices, with hydrophobic and polar residues on opposite sides, suggest a membrane-associated localization for *K. phaffii* Hsp12. The helices are likely partially embedded in the lipid bilayer, as proposed for *S. cerevisiae* Hsp12 (Welker et al., 2010). This interaction between Hsp12 and the lipid bilayer would increase membrane stability and fluidity of *K. phaffii* cells during the stationary phase (Welker et al., 2010). The *0208* gene encodes a hypothetical protein with no homolog in *S. cerevisiae*. Its upregulation in the stationary phase has been previously reported (Dou et al., 2021). We observed that the transcriptional profile of *0208*, with respect to growth phase, carbon source, and temperature, resembles that of *HSP12*. These findings suggest that *0208* may be regulated in a similar manner to *HSP12* and/or its protein product might perform a comparable function to Hsp12. The *S. cerevisiae HSP12* gene is regulated by the transcription factors Msn2/Msn4 (De Groot et al., 2000) and Hsf1 (Nussbaum et al., 2014), consistent with the 10 binding sites for these transcription factors that we identified in the inter-CDS region upstream of the *K. phaffii HSP12* gene. A similar analysis for the *0208* gene revealed 8 binding sites for Msn2/Msn4 and Hsf1, supporting the hypothesis of a similar regulatory mechanism for both *HSP12* and *0208* in *K. phaffii*. The amino acid sequence identity between *K. phaffii* Hsp12 and 0208 is only 16%, which at first glance suggests that they may not be related. However, their three-dimensional structures exhibit a similar topology, including an opposite distribution of hydrophobic and polar residues in their alpha-helices, pointing to a likely functional relationship. While Hsp12 associates with the plasma membrane, 0208 is more likely to interact with the mitochondrial outer membrane. Based on these findings, we hypothesize that the *0208* gene encodes an 18.6 kDa stress-responsive protein whose expression responds to environmental stresses in a manner similar to *HSP12*. The 0208 protein may have a function related to that of Hsp12, possibly at the mitochondrial outer membrane.

The *FDH1* gene (subcluster 1.2) encodes formate dehydrogenase, an enzyme involved in formate detoxification (Sakai et al., 1997). In *Candida boidinii*, *FDH1* is repressed in glucose- or glycerol-grown cells and induced by formate (Sakai et al., 1997; Komeda et al., 2003). *FDH1* downregulation under glucose or glycerol cultures has also been reported in *K. phaffii* (Zhu et al., 2025), which is consistent with our findings. The *FDH1* upregulation that we observed during the stationary phase may result from derepression and induction due to carbon source depletion and formate accumulation. This formate was likely generated from secondary pathway intermediates, such as glycine and serine, *via* the glycine cleavage system (Bysani et al., 2024) and the tetrahydrofolate-mediated one-carbon metabolism (Bustos, Berrios & Fickers, 2024). This response appears more pronounced following growth in glucose, likely due to the higher biomass concentrations achieved compared to growth in glycerol, which may intensify secondary metabolic activity.

*JEN1* and *ADY2*, grouped in subcluster 1.3, encode monocarboxylate permeases primarily involved in lactate and acetate transport (Pacheco et al., 2012). Although their transcriptional patterns suggest a response to carbon source depletion similar to *HSP12* and *0208*, the transcript levels of *JEN1* and *ADY2* were lower than those of *HSP12* and *0208*. They were also consistently higher in glycerol-grown than in glucose-grown cells, indicating that *JEN1* and *ADY2* are likely regulated by a distinct mechanism. In *S. cerevisiae*, *JEN1* promoter activity is repressed at glucose concentrations above 0.3%, remains active in glycerol, and is enhanced by lactate (Chambers, Issaka & Palecek, 2004), which is consistent with our findings in *K. phaffii*. In our experiments, the culture pH dropped below 3.0 during growth, in agreement with previous reports (Chiruvolu et al., 1998). This is likely due to the secretion of organic acids such as lactate and acetate produced during glucose or glycerol metabolism. The accumulation of organic acids likely enhances *JEN1* and *ADY2* transcription during the stationary phase, facilitating their uptake for further metabolism.

*GAPDH* and *FTEII* were classified in the same subcluster (2.1), as expected, since both are regulated by P_GAP_. However, *FTEII* transcript levels were higher than those of *GAPDH*, likely due to the presence of multiple copies of the heterologous *FTEII* gene in the KM71/P_GAP_-FTEII strain, which was selected for *FTEII* overproduction. *GAPDH* encodes a key enzyme involved in the metabolism of glucose and glycerol; therefore, both carbon sources activate P_GAP_, resulting in the expression of the two genes. The higher transcript levels of *GAPDH* and *FTEII* in glucose-grown cells are consistent with previous reports (Waterham et al., 1997; Viader-Salvadó et al., 2024). The slight differences in their transcriptional patterns may be explained by the greater mRNA stability conferred by the *AOX1* transcriptional terminator used in *FTEII* (Viader-Salvadó et al., 2024; Ramakrishnan et al., 2020; Ito et al., 2020).

*ADH2* (subcluster 2.2) encodes the sole alcohol dehydrogenase involved in ethanol metabolism in *K. phaffii* and is active in cells grown in glucose or glycerol (Karaoğlan et al., 2020), which aligns with our findings. It is also induced in the presence of ethanol (Karaoğlan et al., 2020). Our observation of higher transcript levels in glucose-grown cells compared to glycerol-grown cells during the exponential phase is consistent with previous reports (Karaoğlan et al., 2020), considering a key methodological difference. In our experiments, we used the stable reference gene *VPS21*, whereas *ADH2* transcript levels in that study were normalized to *GAPDH*, whose transcriptional activity is known to be higher in glucose-grown than in glycerol-grown cells (Waterham et al., 1997; Viader-Salvadó et al., 2024). The higher transcript levels of *ADH2* during the stationary phase may be attributable to induction by residual ethanol in the culture medium, likely more abundant following growth in glycerol than in glucose.

*TMA10* (classified between clusters 1 and 2) encodes a translation-machinery-associated protein of unknown function (Fleischer et al., 2006). *TMA10* promoter has been described as strong promoter in *K. phaffii*, although with lower strength than P_GAP_ (Dou et al., 2021). In that study, promoter strength was evaluated by measuring intracellular activity of the reporter protein *β*-galactosidase normalized to a P_GAP_-based control strain expressing the same reporter. The results indicated promoter activity during both the exponential and stationary phases, with higher activity in the exponential phase. Additionally, promoter activity of *TMA10* was reported in cells cultivated in both glucose and glycerol, with higher activity observed under glycerol (Dou et al., 2021). Our findings on *TMA10* transcript levels confirm and expand upon these previous observations.

The transcriptional characterization of the selected promoters, based on the transcript levels of their corresponding genes across carbon sources (glucose and glycerol), cell growth phase, and temperatures (24 °C and 30 °C), provides a basis for proposing bioreactor culture strategies using these promoters to drive heterologous gene expression. *K. phaffii* can be cultivated to high biomass concentrations in bioreactors, which is advantageous for promoter-based systems where protein production is growth-associated. This applies to the use of cluster 2 promoters (*GAPDH* and *ADH2*). Moreover, fed-batch strategies with exponential feeding rate profiles, which generate a pseudo-stationary state with a constant specific growth rate (µ), can be used to control promoter activity (Herrera-Estala et al., 2022). To increase volumetric productivity and protein titers, µ should be fine-tuned to balance growth and expression. This would minimize protein misfolding and endoplasmic reticulum-associated degradation (ERAD). In *ADH2* promoter-based strains, the impact of a controlled ethanol feeding strategy could be further investigated.

Although the biomass generated during protein production could be used as a source of single-cell protein and vitamins for feed and food, it often results in large amounts of biomass that become a waste by-product, difficult to repurpose profitably. To increase the product/biomass yield (Y_p/x_) and reduce waste, protein production should be uncoupled from biomass generation. This is possible by using cluster 1 promoters associated with high transcript levels under low µ, as *K. phaffii* remains viable under such conditions (Rebnegger et al., 2024). Specifically, for promoters from subcluster 1.1 (*HSP12* and *0208*), a suitable strategy would involve a batch phase with glucose or glycerol at 28–30 °C for biomass generation, followed by a glucose or glycerol fed-batch phase at 24 °C, with a low feeding rate for cell maintenance and minimal growth. This approach would yield less biomass than that generated by classical P_AOX1_- or P_GAP_-based systems (Bernat-Camps et al., 2023). However, high transcript levels under these promoters do not necessarily imply recombinant protein folding limitations or metabolic burden, since these effects also depend on the physiological context and on the nature of the recombinant protein. Whether this proposed strategy reduces either or both of these limitations remains to be experimentally determined. For *0208* promoter-based strains, using glycerol instead of glucose in the fed-batch phase may reduce costs, as the *0208* gene showed higher transcript levels in glycerol-grown cells and, additionally, glycerol is cheaper than glucose. Furthermore, the stronger downregulation of the *0208* gene during exponential growth compared to *HSP12* may improve the decoupling of growth from protein production.

If the *FDH1* promoter (subcluster 1.2) is used in the expression cassette, the culture strategy would be similar to that for the *0208*- and *HSP12*-promoter-based strains but using glucose instead of glycerol in the fed-batch phase. Moreover, the impact of a controlled formate feeding strategy on heterologous gene expression could be further investigated.

Since *JEN1* and *ADY2* (subcluster 1.3) showed transcriptional patterns consistent with a response to lactate and/or acetate under the acidic conditions generated during glucose or glycerol metabolism, with higher transcript levels observed after glycerol cultures, a suitable strategy for *JEN1*- or *ADY2*-promoter-based strains would start with glycerol batch phase at 28–30 °C, allowing the pH to drop to 3.0. This would be followed by a glycerol fed-batch phase at 30 °C, with a low feeding rate for cell maintenance. Additionally, the impact of lactate or acetate supplementation on heterologous gene expression could be further investigated to increase protein titers. This strategy could support recombinant protein production at pH 3–4, where the main endogenous *K. phaffii* protease released into the culture medium exhibits minimal activity (Salamin et al., 2010). Cultivation strategies at pH 3–4 have also been used to produce a protease in *K. phaffii* that is inactive at acidic pH (Müller et al., 2010), reducing recombinant protein toxicity to host cells.

For a *TMA10* promoter-based strain, a suitable culture strategy would involve an initial glycerol batch phase, followed by a fed-batch phase with an optimized µto enhance heterologous gene expression. Since *TMA10* showed a broad transcriptional profile across both the exponential and stationary growth phases and under both glucose and glycerol, the protein production process would be robust, as recombinant protein titers would exhibit minimal fluctuations under operational variations. Additionally, temperature adjustments during the fed-batch phase could be explored to further improve protein titers. The proposed bioprocess strategies should be considered hypotheses based on transcriptional profiles and would require direct experimental validation in promoter-reporter or production strains.

## Conclusions

A transcriptomic analysis-driven strategy enabled the identification of seven strong promoter candidates in *K. phaffii*. These promoters were identified from endogenous genes showing high transcript levels under specific culture conditions, comparable to or exceeding those of genes regulated by P_AOX1_ or P_GAP_. The corresponding genes showed transcriptional profiles that varied with carbon source, growth phase, and temperature. Two major clusters were identified: one associated mainly with genes upregulated during the stationary phase and the other with genes upregulated during the exponential phase. These findings provide a basis for the rational design of fed-batch fermentation strategies tailored to each promoter when driving heterologous gene expression, although these strategies should be experimentally validated. The *0208* and *HSP12* promoters showed particular potential at low specific growth rates, favoring high recombinant protein titers with reduced biomass accumulation. Overall, these findings expand the genetic toolbox for recombinant protein production in *K. phaffii* and support the development of more flexible and efficient expression systems.

## Supplemental Information

10.7717/peerj.21479/supp-1 Supplemental Information 1 Raw data.

10.7717/peerj.21479/supp-2 Supplemental Information 2 Supplemental Tables.

10.7717/peerj.21479/supp-3 Supplemental Information 3 Growth kinetics of the KM71/P_GAP.-FTEII strain cultivated in glucose or glycerol at 24 °C or 30 °C Points represent the mean ± standard error from data of three independent kinetic experiments. The continuous line represents the fitted data to the integrated solution of the logistic equation.

10.7717/peerj.21479/supp-4 Supplemental Information 4 Hsp12 and 0208 molecular models and helical wheel representations of helices H1-H4 (A) Hsp12 and (B) 0208. Top panels show molecular models; bottom panels show helical wheel representations for helices H1–H4. Helices are shown in red in both molecular models. In the helical wheels, hydrophobic uncharged residues are green, acidic residues red, basic residues blue, and other residues gray.

10.7717/peerj.21479/supp-5 Supplemental Information 5 MIQE checklist.

## Additional Information and Declarations

### Data Availability

The following information was supplied regarding data availability: The raw measurements are available in the Supplemental File. The sequences are available at NCBI and the Supplemental File: PRJNA930494.
