# Supplementary material for: Transcriptomic analysis-driven identification and transcriptional characterization of Komagataella phaffii promoters from highly transcribed endogenous genes across diverse culture conditions
Source: PeerJ. 2026 Jul 21;14:e21479. doi: 10.7717/peerj.21479 (PMC13398393; doi:10.7717/peerj.21479)
Supplement: Supplemental Information 2 [file peerj-14-21479-s002.pdf]

SUPPLEMENTARY INFORMATION

PeerJ

Transcriptomic analysis-driven identification and transcriptional characterization of *Komagataella phaffii* promoters from highly transcribed endogenous genes across diverse culture conditions

Karla Beatriz Fernández-Cano, José María Viader-Salvadó*, Martha Guerrero-Olazarán*
Universidad Autónoma de Nuevo León, UANL, Facultad de Ciencias Biológicas, Instituto de Biotecnología, 66455 San Nicolás de los Garza, N.L., Mexico.

*Corresponding Authors:

Martha Guerrero-Olazarán
José María Viader-Salvadó

Universidad Autónoma de Nuevo León, UANL, Facultad de Ciencias Biológicas,
Instituto de Biotecnología.
Av. Universidad S/N, Col. Ciudad Universitaria, 66455 San Nicolás de los Garza,
Nuevo León, Mexico
e-mail: martha.guerrerool@uanl.edu.mx
e-mail: jose.viadersl@uanl.edu.mx

Table S1. Primer and probe sequences, target accession numbers and amplicon lengths used for RT-qPCR assays.

Primer ID	Sequence	Gene name[a]	Target GenBank accession	Amplicon length (bp)
5qHSP12	GGAGACCCTCACCGATGCTG	*HSP12*	XM_002494021.1	117
3qHSP12	TTGTCGGCCTCGTCCTTACC			
5q0208	GGTGGTTGAGGAGAAGGTGGG	*0208*	XM_002491944.1	123
3q0208	GCACTCTCACTGGCTCCCTC			
5qFDH1	CCCTCAGCCAGCTCCAAAGG	*FDH1*	XM_002493126.1	119
3qFDH1	CGGCATATCTGACCTGGGCG			
5qJEN1	GTCTCCGCCCCATTTCCGAG	*JEN1*	XM_002492622.1	133
3qJEN1	CCGCACTCCATTCTCGTCGT			
5qADY2	TTGGGTCTGTCTGCCTTCGC	*ADY2*	XM_002489758.1	100
3qADY2	AAGCGGCACCAACAACAACG			
5qADH2	CCCAAGCCGCCAGAATTCCA	*ADH2*	XM_002491337.1	145
3qADH2	AGTCCTCCACCAGCACCAGA			
5qTMA10	ACCGACCACTCCCACCTACA	*TMA10*	XM_002493790.1	112
3qTMA10	TTCCCTTTCCGTGGCCGTTC			
5PTFTEII	TCCAAATCACTGACGGTCCAGA	*FTEII*	HM755449.1	118
3PTFTEII	TCACCGTCTTGAGCAACGAACA			
5PTGAPDH	TTCCATCTTCCACTGGTGCT	*GAPDH*	XM_002491300.1	90
3PTGAPDH	TTGGGACACGGAAAGCCAAA			
5qVPS21	GCGGTCAAGCTAGTGCTTCT	*VPS21*	XM_002493580.1	70
3qVPS21	TGCTGACAAAACGTAAAACCA			
**Probe**	**Sequence**			
pFTEII	TTTGGGTTTCGGTTTGGGTCCAGA	*FTEII*	HM755449.1	
pGAPDH	CCAGAATTGAACGGTAAGCTGA	*GAPDH*	XM_002491300.1	

[a]: Gene names are based on the corresponding *S. cerevisiae* homologs, except for PAS_chr2-2_0208, referred to as *0208*, and *FTEII*, which encodes a heterologous β-propeller phytase.

Table S2. Standard curve parameters and amplification efficiency for RT-qPCR assays.

Assay	Slope	Y-intercept	Efficiency (%)
FTEII	-3.545	30.155	91.5
GAPDH	-3.353	29.919	98.7
VPS21	-3.425	26.329	95.9
Universal SYBR Green	-3.521	26.139	92.5

Slopes and y-intercepts were calculated from linear regression of Ct values versus $\log_{10}$ gDNA amount.

Ct values were determined using a fluorescence threshold of 2500 for all assays.

A universal SYBR Green standard curve was used to calculate slope, y-intercept and PCR efficiency for the SYBR Green assays of *HSP12*, *0208*, *FDH1*, *JEN1*, *ADY2*, *ADH2* and *TMA10*. This curve was derived from the average of the *VPS21* standard curve and a SYBR Green standard curve generated for another *K. phaffii* gene (*1033* gene) and previously reported (Robainas-del-Pino et al., 2023). Both standard curves were generated in parallel using the same *K. phaffii* gDNA stock and the same five-point 1:5 serial dilution series; both showed similar slopes and efficiencies.

Table S3. Pairwise comparisons of transcript levels for the evaluated genes showing the effect of the carbon source. The table includes the condition with higher transcript levels, the temperature and cultivation time, and the corresponding *p*-value.

Gene	Result (↑ in)	Temperature (°C)	Time (h)	p-value
HSP12	Glc	24	24	0.048
	Gly	24	12, 48	0.001, 0.041
		30	6, 12, 48	0.000, 0.001, 0.000
0208	Glc	30	12	0.013
	Gly	24	6, 12, 48	0.003, 0.001, 0.031
		30	6	0.017
FDH1	Glc	24	24, 48	0.023, 0.001
		30	24, 48	0.014, 0.001
JEN1	Glc	24	48	0.018
	Gly	24	6, 12, 24	0.005, 0.016, 0.042
		30	6, 12, 24, 48	0.002, 0.003, 0.000, 0.027
ADY2	Gly	24	6, 12, 24, 48	0.014, 0.004, 0.046, 0.007
		30	6, 12, 24, 48	0.021, 0.024, 0.004, 0.003
GAPDH	Glc	24	6, 12, 24, 48	0.006, 0.006, 0.015, 0.003
		30	6, 12	0.024, 0.049
FTEII	Glc	24	6, 12, 48	0.011, 0.005, 0.012
		30	6, 12	0.011, 0.025
	Gly	30	24	0.013
ADH2	Glc	24	6, 12	0.049, 0.037
		30	6, 12	0.049, 0.029
	Gly	24	24	0.002
		30	24, 48	0.005, 0.000
TMA10	Glc	24	12	0.018
	Gly	24	6	0.006
		30	6, 12, 24, 48	0.000, 0.032, 0.018, 0.007

Glc: glucose.
Gly: glycerol.
↑ in: carbon source (Glc or Gly) in which higher transcript levels were observed.

Table S4. Pairwise comparisons of transcript levels for the evaluated genes showing the effect of temperature. The table includes the condition with higher transcript levels, the carbon source and cultivation time, and the corresponding *p*-value.

**Gene**	**Result (↑ in) (°C)**	**Carbon source**	**Time (h)**	**p-value**
HSP12	24	Glc	6, 12, 24, 48	0.000, 0.000, 0.001, 0.001
		Gly	6, 12, 24, 48	0.035, 0.004, 0.049, 0.035
0208	24	Glc	6, 48	0.003, 0.018
		Gly	12, 48	0.007, 0.023
	30	Glc	12	0.041
FDH1	24	Glc	24, 48	0.036, 0.000
		Gly	24, 48	0.024, 0.038
JEN1	24	Glc	48	0.000
	30	Glc	24	0.048
		Gly	6, 12, 48	0.030, 0.012, 0.047
ADY2	24	Glc	24	0.002
	30	Gly	6, 12	0.034, 0.008
GAPDH	24	Glc	6, 12, 24, 48	0.026, 0.048, 0.036, 0.044
		Gly	6	0.003
	30	Gly	48	0.010
FTEII	24	Glc	6, 12, 24, 48	0.039, 0.049, 0.049, 0.019
ADH2	24	Gly	24	0.031
	30	Glc	48	0.037
		Gly	48	0.000
TMA10	24	Glc	24	0.008
	30	Gly	6, 12, 48	0.001, 0.008, 0.013

↑ in: indicates the temperature (24 °C or 30 °C) at which higher transcript levels were observed.

Table S5. Prediction of transcription factor-binding sites (TFBSs) for Msn2/Msn4 and Hsf1 in the upstream inter-CDS region of the *HSP12* gene, using FIMO. A schematic representation of the predicted TFBS positions within the upstream inter-CDS sequence is shown below.

Motif ID	Alt ID	Strand	Start	End	p-value	Matched Sequence
MA0319.2	Hsf1	+	46	52	0.0046	AAAGAAG
MA0319.1	Hsf1	+	46	53	0.0049	AAAGAAGT
MA0342.1	Msn4	+	59	63	0.0007	AGGGG
MA0341.1	Msn2	+	59	63	0.0007	AGGGG
MA0319.1	Hsf1	+	60	67	0.0048	GGGGAAAA
MA0319.2	Hsf1	+	60	66	0.0057	GGGGAAA
MA0342.1	Msn4	+	165	169	0.0007	AGGGG
MA0341.1	Msn2	+	165	169	0.0007	AGGGG
MA0342.1	Msn4	+	166	170	0.0029	GGGGT
MA0342.1	Msn4	-	176	180	0.0022	AGGGT
MA0319.1	Hsf1	-	190	197	0.0038	AAAGAATA
MA0319.2	Hsf1	-	191	197	0.0051	AAAGAAT
MA0342.1	Msn4	-	240	244	0.0029	GGGGT
MA0341.1	Msn2	-	241	245	0.0019	CGGGG
MA0342.1	Msn4	-	241	245	0.0042	CGGGG
MA0319.1	Hsf1	-	299	306	0.0019	ATAGAAAT
MA0319.2	Hsf1	-	300	306	0.0016	ATAGAAA
MA0319.1	Hsf1	-	304	311	0.0090	ATGTAATA
MA0319.1	Hsf1	-	333	340	0.0030	AAGGAAGC
MA0319.2	Hsf1	-	334	340	0.0024	AAGGAAG
MA0319.1	Hsf1	-	337	344	0.0039	AGAGAAGG
MA0319.2	Hsf1	-	338	344	0.0040	AGAGAAG
MA0319.2	Hsf1	+	467	473	0.0079	ATGCAAC
MA0319.1	Hsf1	+	467	474	0.0082	ATGCAACT
MA0341.1	Msn2	+	487	491	0.0019	CGGGG
MA0342.1	Msn4	+	487	491	0.0042	CGGGG
MA0319.1	Hsf1	+	488	495	0.0034	GGGGAATA
MA0319.2	Hsf1	+	488	494	0.0043	GGGGAAT
MA0319.1	Hsf1	-	518	525	0.0008	TTGGAAGA
MA0319.2	Hsf1	-	519	525	0.0012	TTGGAAG
MA0319.1	Hsf1	+	530	537	0.0031	AAGGAAAA
MA0319.2	Hsf1	+	530	536	0.0038	AAGGAAA
MA0319.2	Hsf1	+	581	587	0.0064	CCAGAAG
MA0319.1	Hsf1	+	581	588	0.0070	CCAGAAGT
MA0342.1	Msn4	-	644	648	0.0057	AGGGA
MA0319.1	Hsf1	-	655	662	0.0025	TTAGAAGG
MA0319.2	Hsf1	-	656	662	0.0027	TTAGAAG

Motif ID: unique identifier of the motif from the JASPAR database for fungi, representing a specific transcription factor binding pattern.
Alt ID: alternative identifier of the motif, corresponding to the name of the transcription factor.
Strand: '+' motif matched the forward strand, '-' motif matched the reverse strand.
Start: start position of the motif occurrence.
Stop: end position of the motif occurrence.

p-value: probability that a random sequence of the same length as the motif would achieve a match score equal to or better than the observed alignment at that position.
Matched sequence: region of the sequence matched to the motif.

Schematic representation of predicted TFBS positions in the upstream inter-CDS region of the *HSP12* gene

Sequence of the upstream inter-CDS region of the *HSP12* gene

```
>FN392322.1:1221312-1221976 Pichia pastoris GS115 chromosome
4, complete sequence
TTCTAGTTGTGTTTGTGTTTTGTAACTGTTTAAGTGTGAAAAATAAAAGAAGTTTAGCA
GGGGAAAAGAGTGTCCATTTATACTAAGAAAGGTGTCCTTATAGCAATCTTAGTAAAGT
GATTGCTATGTCATCATTTAGCAATCATGGTTTAGCGAGCAACAATAGGGGTCAAGCAC
CCTGTGCTAAAATATTCTTTCAACTACGAAATTCGCAATAAATGCCTTGTGAAGACAAT
AACACCCCGTTGTACCGCACAATTGCTCTGCAAAATACATCACACCTCACAATTGCTTA
GCAATTTCTATTACATCATAGCTGTGATCGTCACCAAGCTTCCTTCTCTTACTGAAGAC
CGATCTATGACAACACCTGAACACCATCATCATGACACGACCACTATTACACCCACACA
CCAACCACCAATAGATATTTGTTACCACAAATCACCATGTCGTGTTAATTTCAATGCAA
CTAATTTGCCTAATCGGGGAATATTCGCTGATACCCACACAAACCTCTTCCAAGACTAA
GGAAAAACCAGCCATCTTCAAATCTCCCGTCACTCTTTGATCTAACCTGCCAGAAGTGC
TACTTTACTTTATTTTTTGCTTCGACCTCATTTTGTTTGCTTAGAGTGGTGACTCCCTC
ATTCACCTTCTAATCA
```

Gray boxes indicate predicted Msn2/Msn4 TFBSs. Yellow boxes indicate predicted Hsf1 TFBSs.

Table S6 Prediction of transcription factor-binding sites (TFBSs) for Msn2/Msn4 and Hsf1 in the upstream inter-CDS region of the *0208* gene, using FIMO. A schematic representation of the predicted TFBS positions within the upstream inter-CDS sequence is shown below.

Motif ID	Alt ID	Strand	Start	End	p-value	Matched Sequence
MA0319.1	Hsf1	+	89	96	0.0095	GTGTAACA
MA0319.2	Hsf1	+	163	169	0.0044	TTAGAAA
MA0319.1	Hsf1	+	163	170	0.0041	TTAGAAAG
MA0342.1	Msn4	-	226	230	0.0029	GGGGT
MA0341.1	Msn2	-	227	231	0.0026	TGGGG
MA0342.1	Msn4	-	227	231	0.0042	TGGGG
MA0319.2	Hsf1	-	293	299	0.0046	AAAGAAG
MA0319.1	Hsf1	-	292	299	0.0049	AAAGAAGT
MA0319.2	Hsf1	+	332	338	0.0055	TCGGAAT
MA0319.1	Hsf1	+	332	339	0.0045	TCGGAATA
MA0341.1	Msn2	-	358	362	0.0026	TGGGG
MA0342.1	Msn4	-	358	362	0.0042	TGGGG

Motif ID: unique identifier of the motif from the JASPAR database for fungi, representing a specific transcription factor binding pattern.
Alt ID: alternative identifier of the motif, corresponding to the name of the transcription factor.
Strand: '+' motif matched the forward strand, '-' motif matched the reverse strand.
Start: start position of the motif occurrence.
Stop: end position of the motif occurrence.
p-value: probability that a random sequence of the same length as the motif would achieve a match score equal to or better than the observed alignment at that position.
Matched sequence: region of the sequence matched to the motif.

Schematic representation of predicted TFBS positions in the upstream inter-CDS region of the *0208* gene

Sequence of the upstream inter-CDS region of the *0208* gene

```
>FN392320.1:1997016-1997412 Pichia pastoris GS115 chromosome
2, complete sequence
TGATCCTTTAGGTACTTCAGGATGTTTAAGTCATCAAACTGTCCATCAAAGGTAGTATA
GTATTTACCATCTAGATAGTGATGTATGGGTGTAACACAACATTTAAATGTTGTAAATT
AACATTAGGACTGAGTCCGGAGATGCTATTGTCACCTAAATCTATTAGAAAGCACTTCA
GTTATATCATCGATAGAGGTTTGAAGATAAACCTATTGTTGATAAATAACCCCATTACC
CGTTTACGTAGCAAGGTTCAAAAATTTGCTTAGATCGGAGCTAAAAATTCGACTGACTT
CTTTCGAAAATGTGGATTATGCAAGCAACGTTGCTATCGGAATAGTATATAAGGTCGAT
CTGCCCCATTACAAATTGTAAAGCAACAAACATCCTACGCAAA
```

Gray boxes indicate predicted Msn2/Msn4 TFBSs. Yellow boxes indicate predicted Hsf1 TFBSs.

Table S7. Predicted probabilities of subcellular localization for Hsp12 and 0208 proteins using DeepLoc 2.1.

**Localization**	**Probability**	
	**Hsp12**	**0208**
Cytoplasm	0.7315	0.1044
Nucleus	0.4088	0.0951
Extracellular	0.1792	0.0352
Cell membrane	0.2802	0.0345
Mitochondrion	0.3679	0.9298
Plastid	0.0674	0.0018
Endoplasmic reticulum	0.2846	0.0179
Lysosome/Vacuole	0.2293	0.0334
Golgi apparatus	0.1961	0.0166
Peroxisome	0.0356	0.0022

Table S8. Predicted probabilities of membrane association type for Hsp12 and 0208 proteins using DeepLoc 2.1.

**Membrane association**	**Probability**	
	**Hsp12**	**0208**
Peripheral	0.5526	0.579
Transmembrane	0.1281	0.174
Lipid anchor	0.0717	0.135
Soluble	0.6235	0.614
