# Supplementary figures and images for: Transcriptomic analysis-driven identification and transcriptional characterization of Komagataella phaffii promoters from highly transcribed endogenous genes across diverse culture conditions
Source: PeerJ. 2026 Jul 21;14:e21479. doi: 10.7717/peerj.21479 (PMC13398393; doi:10.7717/peerj.21479)

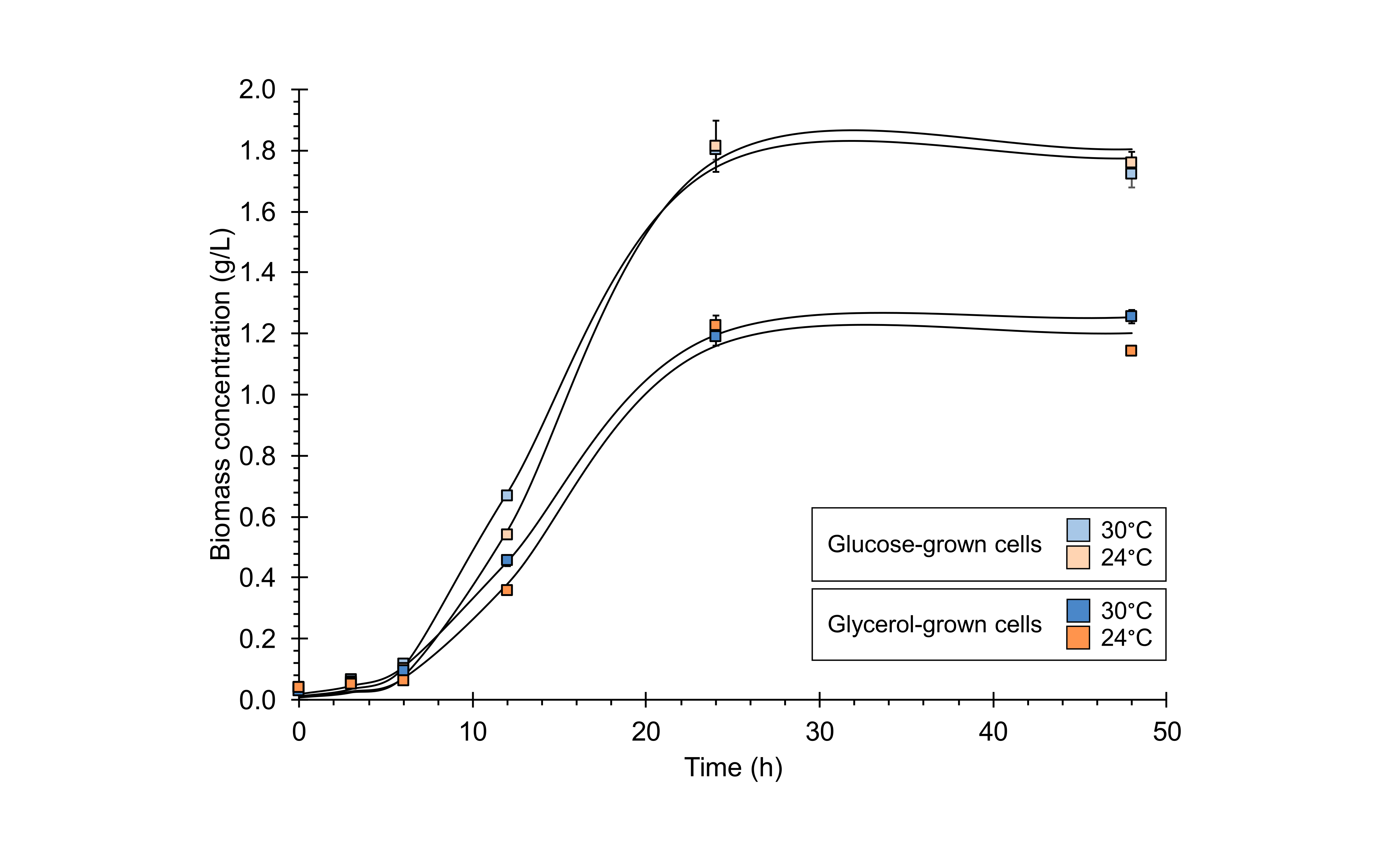

Supplement: Supplemental Information 3 — Points represent the mean ± standard error from data of three independent kinetic experiments. The continuous line represents the fitted data to the integrated solution of the logistic equation. [file peerj-14-21479-s003.png]

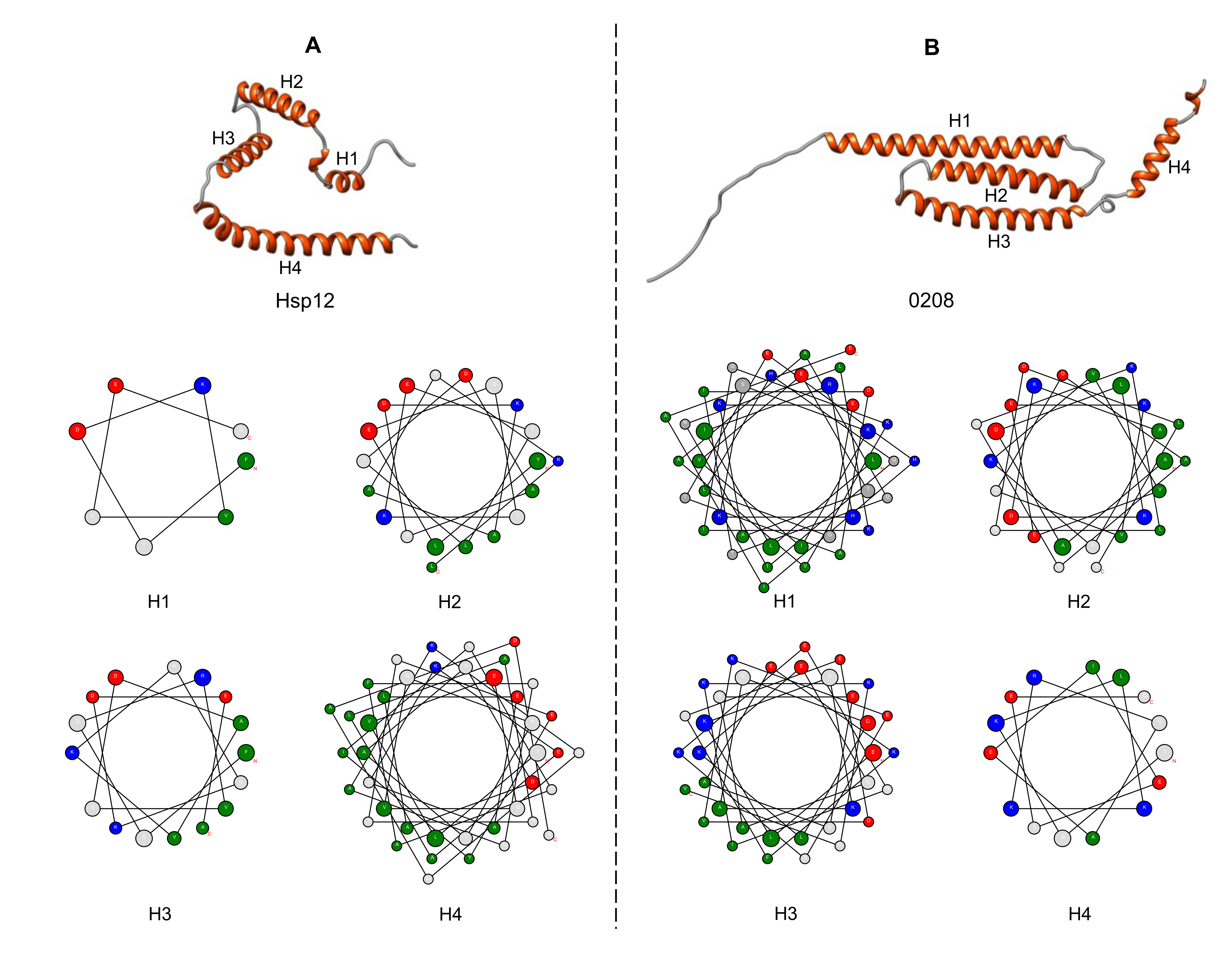

Supplement: Supplemental Information 4 — (A) Hsp12 and (B) 0208. Top panels show molecular models; bottom panels show helical wheel representations for helices H1–H4. Helices are shown in red in both molecular models. In the helical wheels, hydrophobic uncharged residues are green, acidic residues red, basic residues blue, and other residues gray. [file peerj-14-21479-s004.png]
